# The circRNA–microRNA code: emerging implications for cancer diagnosis and treatment

**DOI:** 10.1002/1878-0261.12468

**Published:** 2019-02-18

**Authors:** Lorena Verduci, Sabrina Strano, Yosef Yarden, Giovanni Blandino

**Affiliations:** ^1^ Unit of Oncogenomic and Epigenetic IRCCS Regina Elena National Cancer Institute Rome Italy; ^2^ Department of Biological Regulation Weizmann Institute of Science Rehovot Israel

**Keywords:** cancer, circRNA code, circular RNAs, microRNAs, patients

## Abstract

Circular RNAs (circRNAs) comprise an emerging new class of endogenous RNAs expressed abundantly by the eukaryotic transcriptome. They are characterized by a covalently closed loop structure, resulting in RNA molecules that are more stable than linear RNAs. A growing number of studies indicate that circRNAs play critical roles in human diseases and show great potential as biomarkers and therapeutic targets. The molecular events determined by circRNA activity, the circRNA code, involve other types of noncoding RNA molecules, primarily microRNAs, long noncoding RNAs, and RNA‐binding proteins. Herein, we mainly focus on the circRNA–microRNA code, showing how this relationship impacts the regulation of gene expression in cancer. The emerging roles for circRNAs in oncogenic pathways highlight new perspectives for the detailed molecular dissection of cancer pathogenesis and, at the same time, offer new opportunities to design innovative therapeutic strategies. Here, we review recent research advancements in understanding the biogenesis, molecular functions, and significance of circRNAs in cancer diagnosis and treatment.

**Abbreviations**
3′‐UTR: 3′‐untranslated region
Alu elements: Arthrobacter luteus elements
ATC: anaplastic thyroid carcinoma
CCND3: cyclin D3
CDR1as: cerebellar degeneration‐related protein 1 transcript
ceRNA: competing endogenous RNA
CFS: human cell‐free saliva
circRNAs: circular RNAs
ciRNAs: intronic circRNAs
CRC: colorectal cancer
ecircRNAs: exonic circRNAs
EGF: epidermal growth factor
ElciRNAs: exon–intron circRNAs
EMT: epithelial‐to‐mesenchymal transition
ESCC: esophageal squamous cell carcinoma
HCC: hepatocellular carcinoma
HNSCC: head and neck squamous cell carcinoma
*hTERT*: human telomerase reverse transcriptase
MBL: splicing factor muscleblind
miRNA: microRNA
MRE: miRNA response element
QKI‐5: RNA‐binding protein quaking‐5
RBP: RNA‐binding protein
RNA pol II: RNA polymerase II
RNA‐seq: RNA sequencing technology
rRNA: ribosomal RNAs
Sry: testis‐determining gene
tRNA: transfer RNAs

## 1. Introduction

Circular RNAs (circRNAs) are an abundant class of endogenous RNAs, which have recently been rediscovered and re‐evaluated for their important roles in the regulation of gene expression (Jeck and Sharpless, 2014). These covalently closed circular RNA molecules were previously considered as viroids (Sanger *et al*., 1976), Hepatitis delta virus molecules (Kos *et al*., 1986) and splicing error result (Cocquerelle *et al*., 1993). Many progresses on circRNAs characterization have been made recently. However, full comprehension of their biogenesis and function remains elusive. circRNAs partly originate from protein‐coding genes and can be represented by one exon or by a combination of several exons. circRNAs are characterized by the lack of 3′ poly(A) tails and 5′ end caps. Due to the absence of accessible ends, circRNAs are resistant to exonuclease RNase R and more stable than the corresponding linear RNA isoforms (Jeck and Sharpless, 2014; Memczak *et al*., 2013). Nevertheless, circRNAs are usually expressed at lower levels than their host genes, although it is not the rule (Enuka *et al*., 2016; Salzman *et al*., 2012, 2013). Indeed, Liang *et al*. (2017) showed that the levels of Drosophila circRNAs strongly increase upon inhibition or slowing of multiple canonical pre‐mRNA processing events. This condition is promoted by the long half‐lives of circular RNAs.

Recently, circRNAs emerged for their association with human diseases and in particular with cancer, suggesting their possible use as new biomarkers and therapeutic targets (Chapman *et al*., 2012; Chen *et al*., 2017; Li *et al*., 2015b,2015c; Memczak *et al*., 2015).

## 2. Biogenesis of circRNAs

**Figure 1:**
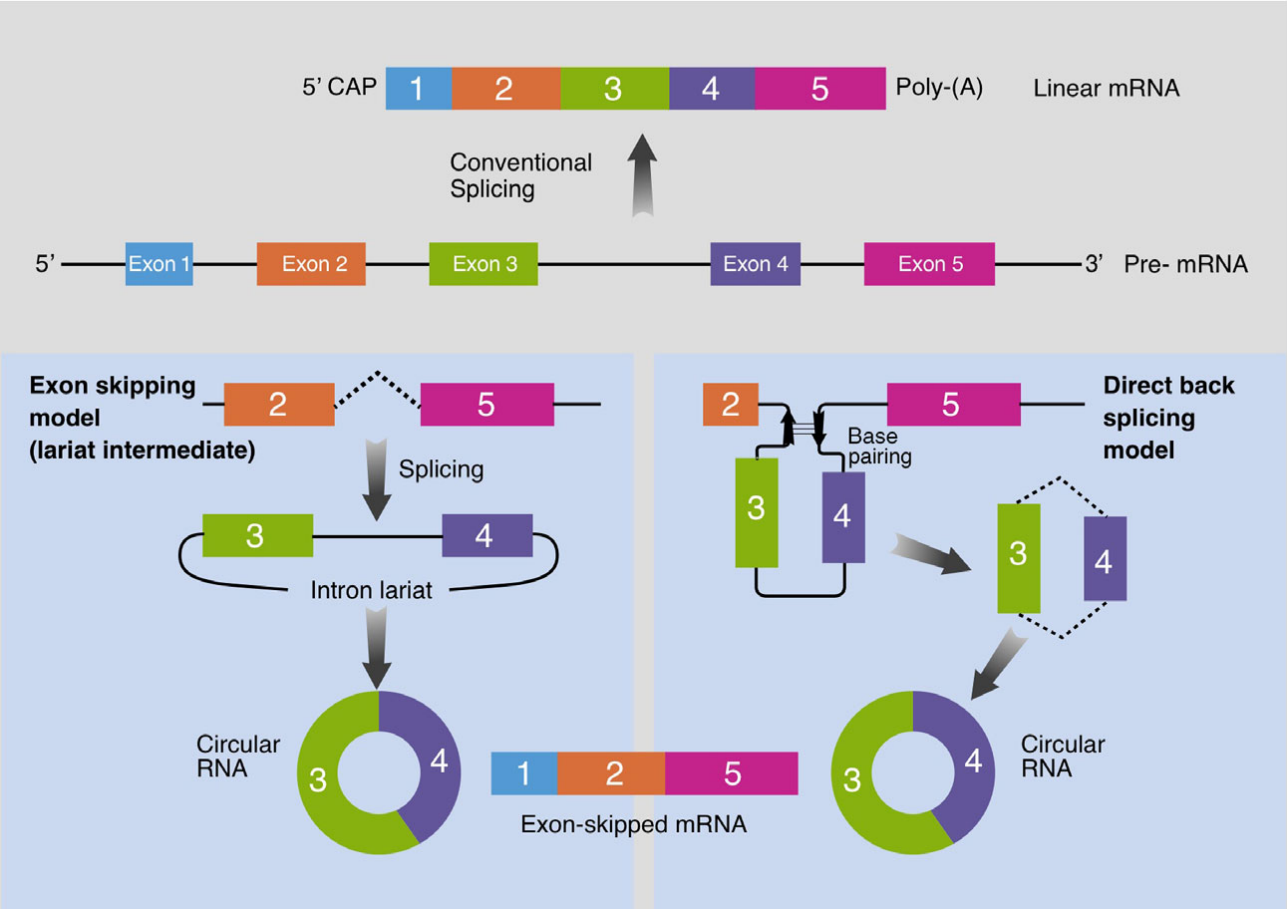
Models proposed to explain the biogenesis of circRNAs. A schematic representation of the two models that generate circRNAs: left, the exon skipping or lariat intermediate model; right, the direct back‐splicing model.

circRNAs can be distinguished based on their origin. Although the exonic circRNAs (ecircRNAs) represent the most abundant part, circRNAs can also derive from intronic, antisense, 5′‐ or 3′‐untranslated, and intergenic genomic regions (Salzman *et al*., 2012). circRNAs comprised of exonic sequences are produced by a mechanism called ‘back‐splicing’ where a downstream 5′ splice site of an exon is joined to an upstream 3′ splice site of another exon, involving single or multiple exons (Ashwal‐Fluss *et al*., 2014; Ivanov *et al*., 2015; Jeck and Sharpless, 2014; Liang and Wilusz, 2014; Starke *et al*., 2015; Zhang *et al*., 2014) (Fig. 1). The result of this mechanism is a covalently closed circular transcript and an alternatively spliced linear RNA with skipped exons.

Most circRNAs are hosted by protein‐coding genes, indicating that RNA polymerase II (RNA pol II) is responsible for their transcription and that their biogenesis is mediated by spliceosomes. Indeed, Ashwal‐Fluss *et al*. (2014) showed that the circRNAs are generated cotranscriptionally competing with canonical pre‐mRNA splicing and consequently affecting the rate of canonical gene expression (Ashwal‐Fluss *et al*., 2014). Later, Starke *et al*. (2015) showed that the exon back‐splicing mechanism requires the canonical spliceosomal machinery.

There are two current models explaining the formation of circRNAs: the exon skipping or lariat intermediate model, and the direct back‐splicing model (Chen and Yang, 2015) (Fig. 1). In the exon skipping or lariat intermediate model, canonical splicing occurs first, generating a linear RNA with skipped exons. The long intron lariat containing these skipped exons is generated determining the production of circRNA by back‐splicing. In the direct back‐splicing model, processing starts with back‐splicing determining a circRNA and an exon–intron(s)–exon intermediate, which can be processed in a linear RNA with skipped exons (Chen and Yang, 2015; Jeck *et al*., 2013; Memczak *et al*., 2013) (Fig. 1).

The mechanism of circular RNA biogenesis is associated with Alu elements (Arthrobacter luteus elements), identified upstream and downstream of the flanking introns of circularized exons (Jeck *et al*., 2013). Moreover, it has been shown that Alu elements can compete between them generating multiple circRNAs from a single gene locus (Jeck *et al*., 2013; Salzman *et al*., 2013; Zhang *et al*., 2014).

The biogenesis of circRNAs is also regulated by splicing factors and RNA‐binding proteins (Ashwal‐Fluss *et al*., 2014; Conn *et al*., 2015). The splicing factor muscleblind (MBL) can promote circRNA biogenesis. MBL promotes the circularization of the circular RNA circMBL, binding to the introns flanking the circRNA generated from the second exon of its own RNA (Ashwal‐Fluss *et al*., 2014). Interestingly, the circRNA also contains binding sites for its parental gene MBL (Ashwal‐Fluss *et al*., 2014). This is an example of how a splicing factor can regulate the production of circRNAs. The MBL example also provides evidence that circRNAs are generated cotranscriptionally, competing with the canonical splicing of pre‐mRNA.

In analogy, the RNA‐binding protein quaking‐5 (QKI‐5) promotes circRNA biogenesis during epithelial‐to‐mesenchymal transition (EMT) binding the introns flanking the circRNA‐forming exons (Conn *et al*., 2015). QKI‐5, the most abundant isoform, has been found to act as a tumor suppressor in cancer (Zhao *et al*., 2014). For example, in prostate cancer, QKI‐5 is downregulated as a consequence of hypermethylation of the respective promoter. In addition, QKI‐5 inhibits cell proliferation and promotes cancer cell apoptosis (Zhao *et al*., 2014). QKI‐5 has been found to be downregulated also in lung cancer, and this associates with poorer patient's prognosis. Interestingly, the tumor suppressor action of QKI‐5 relates to an ability to regulate alternative splicing of NUMB, a key target of the Notch signaling pathway (Zong *et al*., 2014). Under nontumoral conditions, the exclusion of exon 12 of NUMB's pre‐mRNA determines the block of Notch. In a context where QKI‐5 is downregulated, as it is in cancer, NUMB is expressed with the inclusion of the exon 12, resulting in an isoform able to activate Notch (Zong *et al*., 2014).

The tumor suppressor role of QKI‐5, combined with its capability to promote circRNA biogenesis during EMT, suggests a central role for QKI‐5 in the regulation of circRNAs biogenesis in cancer. Indeed, EMT plays an important role in promoting cancer metastasis (Conn *et al*., 2015; Tsai and Yang, 2013; Zhao *et al*., 2014; Zong *et al*., 2014).

Further investigations will be necessary to completely elucidate the mechanism and regulation of circRNA biogenesis.

## 3. Known mechanisms of action of circRNAs

Different computational pipelines have been developed in order to identify and quantify circRNAs based on data of the high‐throughput RNA sequencing technology (RNA‐seq) (Enuka *et al*., 2016; Guo *et al*., 2014; Jeck *et al*., 2013; Salzman *et al*., 2012). Although all approaches employed demonstrated generation of circRNAs from several human loci, their mechanism of action and biological importance remain controversial. Guo *et al*. (2014) identified thousands of circRNAs from a large variety of human cell lines. They argued that, with a few exceptions, circRNA did not show the properties of microRNA (miRNA) sponge and they could be only alternative isoform of their respective primary transcript (Guo *et al*., 2014). Congruent with this line of evidence, while stimulation of mammary cells with the epidermal growth factor (EGF) leads to dynamic changes in the abundance of many coding and noncoding RNA molecules and culminates in the acquisition of a robust migratory phenotype, circRNAs of EGF‐stimulated mammary cells are stably expressed, while mRNAs and miRNAs change within minutes (Enuka *et al*., 2016). The capability to act as miRNA sponge was one of the first mechanisms of action attributed to circRNAs (Hansen *et al*., 2013). Recently, the evidences for circRNAs acting as miRNA sponge are growing and it seems to be one of the most favorite mechanisms in cancer. Several groups working independently showed that circRNAs exert important roles in gene expression regulation by different mechanisms. These RNA molecules can regulate alternative splicing (Ashwal‐Fluss *et al*., 2014; Zhang *et al*., 2014) and sequester RNA‐binding proteins and ribonucleoprotein complexes (Hansen *et al*., 2013; Salzman *et al*., 2012). In addition, circRNAs can be translated and bind in trans to other RNA sequences (Jeck and Sharpless, 2014). A list of circRNAs involved in cancer and discussed here is shown in Table 1.

**Table 1:** List of circRNAs involved in cancer and discussed in this review

Name of circRNAs	Type of cancer	Expression	Targets	References
CDR1	Hepatocellular carcinoma	Up	miR‐7	Yu *et al*. (2016)
Sry	Anaplastic thyroid carcinoma	Up	miR‐138	Hansen *et al*. (2013)
circ‐ITCH	Esophageal squamous cell carcinoma	Down	miR‐17, miR‐214, miR‐7	Li et al. (2015a)
circHIPK3	Liver cancer	Up	miR‐124	Zheng et al. (2016)
circ‐000984	Colorectal cancer	Up	miR‐106b	Xu et al. (2017)
circ‐TTBK2	Human malignant glioma	Up	miR‐217	Zheng *et al*. (2017)
circPVT1	Head and neck squamous cell carcinoma	Up	miR‐497‐5p	Verduci *et al*. (2017)
	Gastric cancer	Up	miR‐125 family	Chen *et al*. (2017)
circ‐PABPN1	Cervical carcinoma	Down	RNA‐binding protein HuR	Abdelmohsen *et al*. (2017)
circ‐Foxo3	Different cancer cell lines	Down	p21‐CDK2	Du *et al*. (2016)
circ‐Amotl1	Breast cancer	Up	c‐myc	Yang *et al*. (2017)
circ_002059	Gastric cancer	Down	Unknown	Li *et al*. (2015b)

### 3.1. circRNAs can act as miRNA sponges

**Figure 2:**
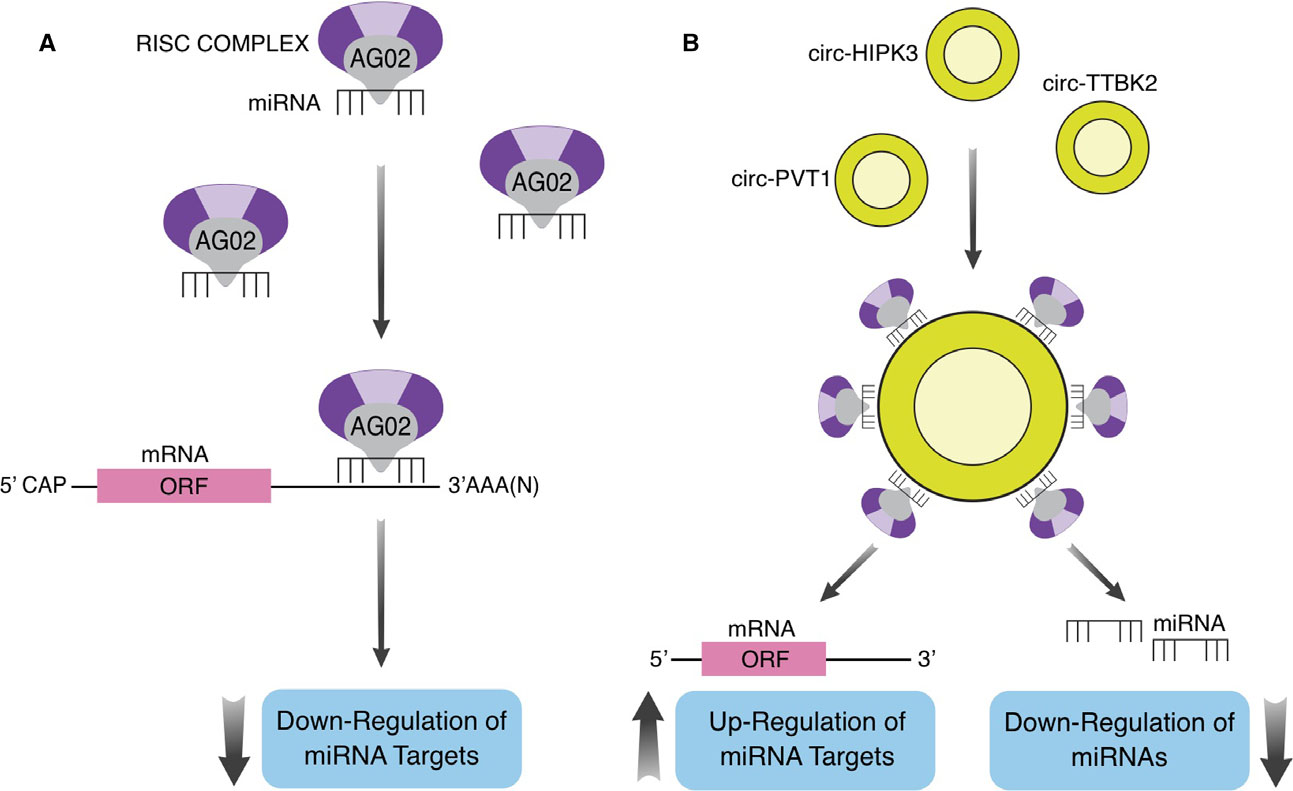
Circular RNAs can affect the miRNA activity. (A) Schematic representation of miRNA mechanism of action. (B) Schematic representation of circRNAs acting as miRNAs sponge. Three examples of sponge‐like circRNAs are shown.

Acting as competing endogenous RNA (ceRNA), or miRNA sponge, circRNAs can bind miRNAs through a miRNA response element (MRE) and negatively regulate their activity (Memczak *et al*., 2013). Since the length of exon circRNAs goes from 100 nt to more than 4 kb (Lasda and Parker, 2014), the potential number of MREs is variable and relates to the length of the circRNAs, as well as to its nucleotide sequence (Fig. 2).

The miRNAs are an abundant class of small noncoding RNAs approximately 22 nucleotides long. They act as negative regulators of gene expression at the post‐transcriptional level, by means of binding their target mRNAs through imperfect base pairing with the respective 3′‐untranslated region (3′‐UTR) (Lewis *et al*., 2005) (Fig. 2).

As miRNA sponges, circular RNAs can function as oncogenes or as tumor suppressor genes. We describe below some significant examples of circRNAs acting as miRNA sponges in a wide range of cancers.

One of the first identified human circRNAs acting like miRNA sponges was the antisense to the cerebellar degeneration‐related protein 1 transcript (CDR1as), also known as ciRS‐7. CDR1as is able to bind miR‐7 using more than 70 conserved miRNA target sites, a miRNA sponge first identified in zebrafish neuronal tissues. CDR1as’ expression in zebrafish impairs midbrain development, a phenotype similar to morpholino miR‐7 knockdown. Later, CDR1as was found to be upregulated in hepatocellular carcinoma, where its expression level was inversely correlated with that of miR‐7 (Yu *et al*., 2016). miR‐7 plays an important role in the biology of many cancers, including hepatocellular carcinoma (Fang *et al*., 2012), tongue carcinoma (Jiang *et al*., 2010), schwannoma (Saydam *et al*., 2011), gastric carcinoma (Kong *et al*., 2012), and cervical cancer (Liu *et al*., 2013). It follows that the action of CDR1as can have significant implications in many types of cancers.

The circular transcript of the testis‐determining gene (Sry) was discovered many years ago, but only recently its role as a miRNA sponge has been clarified (Capel *et al*., 1993). Sry sponges miR‐138 due to its 16 target sites for this miRNA (Hansen *et al*., 2013). Sry seems to act in cancer like an oncogene targeting the tumor suppressor miR‐138. miR‐138 is significantly downregulated in anaplastic thyroid carcinoma (ATC), where it regulates cyclin D3 (CCND3), as well as in hepatocellular carcinoma (HCC), where it regulates the human telomerase reverse transcriptase (*hTERT*) (Mitomo *et al*., 2008; Wang *et al*., 2012).

In addition to sponging a single type of miRNA, some circRNAs may regulate more than one miRNA. This seems to be the case with the circular RNA circ‐ITCH. circ‐ITCH is derived from several exons of the E3 ubiquitin (Ub) protein ligase (ITCH). Li *et al*. (2015a) showed that circ‐ITCH is downregulated in esophageal squamous cell carcinoma (ESCC) and it is able to influence the expression level of its host gene, ITCH (Li *et al*., 2015a). circ‐ITCH acts as a miRNA sponge in ESCC by binding with miR‐17, miR‐214, and miR‐7 and thereby increases the level of the linear form ITCH's mRNA. The latter shares with circ‐ITCH the same miRNAs binding sites, which are located on its 3′‐untranslated region (UTR). ITCH is able to suppress the action of the canonical Wnt pathway, which plays an important role in carcinogenesis of many cancer subtypes (Gao and Chen, 2010). Thus, circ‐ITCH plays an antitumor role in ESCC by indirect inhibition of Wnt signaling.

Zheng *et al*. (2016) and colleagues generated ribosomal‐depleted RNA sequences and found many circRNAs are differently expressed between normal and cancerous tissues. The authors focused on the circular RNA circHIPK3, which was significantly upregulated in liver cancer compared with matched normal tissues. circHIPK3 derives from HIPK3 mRNA, and differently from its linear isoform, it acts as a cell growth modulator in human cells. The oncogenic role of circHIPK3 is exerted by directly binding to and inhibiting the tumor suppressor miR‐124 (Zheng *et al*., 2016).

Xu *et al*. (2017) showed that the circular RNA hsa_circ_000984 is upregulated in colorectal cancer (CRC) and is able to affect cell proliferation, migration, and invasion *in vitro* and tumor formation *in vivo*. hsa_circ_000984 acts as a miRNA sponge that binds with miR‐106b. Interestingly, the authors found that the host gene of the hsa_circ_000984, the *CDK6* gene, is a target of miR‐106b. In its action as a miR‐106b sponge**,** hsa_circ_000984 shows the capability to regulate the CRC carcinogenesis through an indirect regulation of its host *CDK6* gene within a circRNA–miRNA–mRNA network (Xu *et al*., 2017).

Zheng *et al*. (2017) found that circ‐TTBK2, but not linear TTBK2, is upregulated in human malignant glioma. circ‐TTBK2 gains oncogene features by binding with miR‐217, which is usually downregulated in glioma tissues and cell lines. circ‐TTBK2 increases HNF1β expression in cancer, acting as miR‐217 sponge, and as a result, miR‐217 cannot negatively regulate HNF1β by targeting its 3′‐UTR. HNF1β is known to activate the transcription of oncogene Derlin‐1 binding its promoter region (Zheng *et al*., 2017).

Recently, we showed that the circular RNA circPVT1 acts as an oncogene in head and neck squamous cell carcinoma (HNSCC). Moreover, the complex containing YAP, TEAD, and a mutant form of p53 transcriptionally enhances its expression (Verduci *et al*., 2017). The phenotype induced by circPVT1 modulation in cancer cell lines appeared to be independent from its host gene, *PVT1*. circPVT1 is able to bind miR‐497‐5p by a specific binding site impairing its tumor suppressor activity. As a result, circPVT1 overexpression in HNSCC determines the upregulation of the genes aurka, mki67, and bub1, all of which are targets of miRNA‐497‐5p involved in cell proliferation (Verduci *et al*., 2017).

### 3.2. The circRNA–microRNA code in tumorigenesis

MicroRNAs (miRNAs) comprise a family of small noncoding, single‐stranded RNAs involved in post‐transcriptional gene silencing (Filipowicz *et al*., 2008; Lewis *et al*., 2005). miRNAs play important roles in a wide range of biological processes, and they are often dysregulated in human cancer (Peng and Croce, 2016).

The mechanisms of miRNAs dysregulation are different. They include alterations in genomic miRNA copy numbers and gene locations leading to amplification or deletion of miRNA genes, alterations of the transcriptional control of miRNAs by transcription factors that play key role in cancer, dysregulated epigenetic changes, such as DNA methylation and histone acetylation of miRNA genes, and defects in the miRNA biogenesis machinery (Peng and Croce, 2016).

Compelling evidences showed that the same miRNAs might act either as tumor suppressors or as oncogenes, depending on the cellular context (Peng and Croce, 2016; Shenouda and Alahari, 2009). Hence, it is important to consider each miRNA in the context of specific diseases of interest.

The detection of the tumor in the early phase of its development is important to increase the chances of successful treatments. The identification of molecules that can be used as long‐term predictors is crucial not only for increasing the survival of patients but also for cancer prevention.

The recent discovery of the circRNA–microRNA code, in which the two types of RNA molecules interact with one another to determine the regulation of gene expression, is a promising field of study for early detection and prognosis of cancer. Our previous studies described miRNAs that can serve as long‐term predictors of postmenopausal breast cancer risk, that is, miR‐145‐3p, miR‐145‐5p, and miR‐513a‐5p (Muti *et al*., 2014, 2018). To the best of our knowledge, there are no studies describing the role of circRNAs as long‐term predictors, but this role is likely to be uncovered. Since circRNAs act as miRNA sponges, it is plausible that miRNA dysregulation in cancer is due, at least in part, to the action of circRNAs, in addition to other known mechanisms.

### 3.3. circRNAs can interact with proteins

The RNA‐binding protein (RBP) HuR can bind several circRNAs. In particular, it shows strong associations with the circular RNA circ‐PABPN1 in human cervical carcinoma HeLa cells (Abdelmohsen *et al*., 2017). HuR is responsible for the translation rate of the *PABPN1* gene. High levels of circ‐PABPN1 are able to sequester HuR and suppress HuR binding to the PABPN1 mRNA. Consequently, circ‐PABPN1 reduces PABPN1 translation and lowers HeLa cell proliferation. This is an example of how circRNAs can affect the translation of their corresponding linear mRNAs competing with them for a RBP (Abdelmohsen *et al*., 2017).

Du *et al*. (2016) demonstrated that the circular RNA circ‐Foxo3 is able to inhibit cell proliferation and cell cycle progression thanks to a ternary complex with CDK2 and p21. Although circ‐Foxo3 acts as a tumor suppressor, like its host gene *Foxo3*, the levels of the circular RNA are not correlated with the level of the corresponding linear mRNA. The circ‐Foxo3‐p21‐CDK2 complex repressed CDK2–cyclin A/E complex formation resulting in block of cell cycle progression (Du *et al*., 2016).

circ‐Amotl1 is highly expressed both in patient tumor samples and in breast cancer cell lines, where it acts as an oncogene promoting cancer growth (Yang *et al*., 2017). circ‐Amotl1 interacts with c‐myc increasing the retention of nuclear c‐myc, promoting c‐myc stability, and upregulating c‐myc targets. Indeed, the complex circ‐Amotl1/c‐myc increases the affinity of c‐myc binding to the promoters of the genes *HIF‐1g*,* Cdc25a*,* ELK‐1*, and *JUN* (Yang *et al*., 2017). Once again, this proves how the function of circular RNAs and the function of the respective host genes are ruled by different mechanisms even if both molecules promote cancer progression.

### 3.4. circRNAs as transcriptional regulators

Intronic circRNAs (ciRNAs) and exon–intron circRNAs (ElciRNAs) represent a peculiar class of circRNAs. In the study of ciRNAs, a distinction between ciRNAs and lariat intronic RNA should be made carefully.

It has been shown that some ciRNAs and ElciRNAs can act as transcriptional regulators (Li *et al*., 2015d; Zhang *et al*., 2013). The intron circRNA ci‐ankrd52 was found mainly in the nucleus and enriched at its sites of transcription. ci‐ankrd52 is able to regulate its parent gene expression by modulating RNA polymerase II's elongation activity. The mechanism of action of this and other intronic circRNAs in the nucleus suggests a new *cis*‐regulatory role for circular intronic transcripts in expression of their parental coding genes (Zhang *et al*., 2013).

In similarity to ciRNAs, also the ElciRNAs are mainly localized to the nucleus. ElciRNAs are RNA molecules in which the exons are circularized with introns ‘retained’ in between the exonic sequences. Similarly to ciRNAs, ElciRNAs can promote expression of their parental genes by a cis‐regulatory mechanism. A specific feature of ElciRNAs, such as circEIF3J and circPAIP2, is their ability to regulate their parental genes due to a specific RNA–RNA interaction between U1 snRNA and ElciRNA (Li *et al*., 2015d).

## 4. circRNAs as products of the RNA‐mediated epigenetic regulation of gene expression

The field of epigenetics research has been focused for decades on DNA and histone modifications, giving marginal attention to RNA modifications, mostly confined to transfer RNAs (tRNA) and ribosomal RNAs (rRNA). Recently, the young field of epitranscriptomics, considered as any chemical modification of RNA, is emerging for its important role in cancer biology, hence attracting a strong research interest. Considering epitranscriptomics from a wider perspective, RNA modifications can be classified in two groups: reversible modifications, such as the different RNA methylations, and nonreversible modifications, such as editing and splicing (Esteller and Pandolfi, 2017). According to this classification, circRNAs may be allocated to the nonreversible modifications of RNA (Esteller and Pandolfi, 2017). In miRNAs, one of the most characterized targets of circRNAs up to now, chemical modifications like adenosines in the form of m^6^A, is important for the control of miRNA expression level (Esteller and Pandolfi, 2017). In particular, the m^6^A was found at the junction between the hairpin stem and the flanking single‐stranded RNA regions of pri‐miRNAs (Alarcón *et al*., 2015b; Esteller and Pandolfi, 2017). The depletion of this modification diminished DGCR8 binding to pri‐miRNAs determining a block in miRNA biogenesis and failure of mature miRNA production (Alarcón *et al*., 2015a).

From an epigenetic point of view, circRNAs by themselves can be considered an outcome of RNA epigenetic regulation. Depending on the role played by miRNA/miRNAs targeted in cancer, circRNAs can have a role of tumor suppressor or oncogene. The study of RNA modification machinery in cancer, in particular regarding the circRNA–microRNA code, is an interesting and promising area of research.

## 5. circRNAs as biomarkers

**Figure 3:**
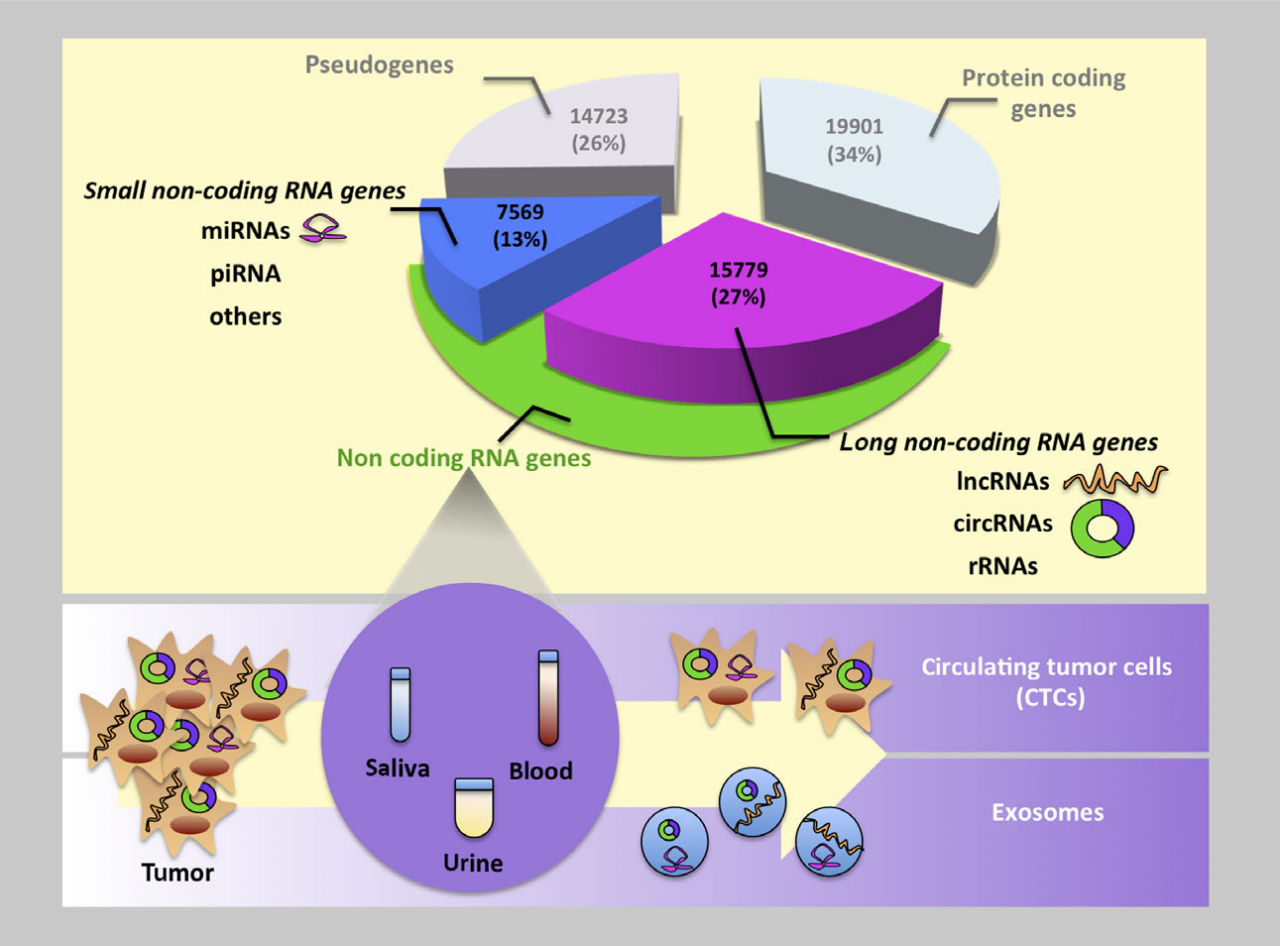
Cancer biomarkers and gene expression signatures. The noncoding part of the genome plays an important role in the regulation of gene expression in cancer (Harrow *et al*., 2012).

The unique biochemical features of circRNAs made this class of RNA molecules promising cancer biomarkers. Indeed, circRNAs are highly stable and relatively abundant molecules showing cell‐, tissue‐, and developmental‐stage‐specific patterns of expression. In addition, circRNAs are highly conserved among the species and resistant to RNase R activity. Moreover, circRNAs exist in exosomes and they have been found in accessible body fluids like saliva, plasma, and blood (Fig. 3).

Thousands of circRNAs have been detected in human peripheral whole blood by RNA sequencing (Memczak *et al*., 2015). Interestingly, in many cases circRNA expression levels are higher than the levels of the corresponding linear RNA isoform (Memczak *et al*., 2015).

The circ_002059 has been found significantly downregulated in gastric cancer tissues compared with paired adjacent nontumor tissues (Li *et al*., 2015b), showing putatively a tumor suppressor activity. Moreover, the expression level of circ_002059 has been correlated with distal metastasis, TNM stage, gender, and age, showing the importance of this circRNA as a prognostic marker for gastric carcinoma (Li *et al*., 2015b). Another circRNA involved in gastric cancer is circPVT1. This circular RNA was found to be upregulated in gastric cancer tissues, suggesting an oncogenic activity (Chen *et al*., 2017). Indeed, the level of circPVT1 has been correlated with overall survival and disease‐free survival of patients. circPVT1 acts like an onco‐circular RNA in gastric carcinoma by acting as a sponge for members of the miR‐125 family (Chen *et al*., 2017). In the same line, we recently found that circPVT1 is upregulated in tumor tissues of HNSCC patients. Patients with high circPVT1 levels have poorer overall survival in comparison with those exhibiting low circPVT1 levels (Verduci *et al*., 2017). Multivariable analysis confirmed the correlation between high circPVT1 levels and reduced overall survival [43]. The onco‐circular RNA circPVT1 exerts its role in HNSCC by acting as a sponge for the miRNA‐497‐5p, a tumor suppressor miRNA associated with p53 mutations in several cancers (Guo *et al*., 2013; Huang *et al*., 2015; Li *et al*., 2011; Verduci *et al*., 2017; Wang *et al*., 2015). It is clear that even small circular RNA, such as circPVT1, which has a length of approximately 400 bp and comes from a single exon of the long noncoding *PVT1* gene, can bind more than one miRNA and act as a gene expression regulator in cancer.

Exosomes are small membrane vesicles released by most cell types with an approximate size of 40–100 nm. They are known for their specific cargos of proteins, mRNAs, and miRNAs, with the potential to be used as biomarkers for diagnosis of human diseases (Li *et al*., 2015c; Pant *et al*., 2012). Li *et al*. (2015c) showed for the first time the presence of circRNAs in exosomes derived from cancer cells using RNA sequencing (Li *et al*., 2015c). Interestingly, circRNAs in exosomes, named exo‐circRNAs, are more abundant in exosomes compared to the cytoplasm of producer cells. In addition, exo‐circRNAs have been identified in serum of healthy donors (Li *et al*., 2015c). The presence of both circRNAs and miRNAs in exosomes suggests the presence of miRNA sponging mechanisms inside these vesicles.

circRNAs have been found also in human cell‐free saliva (CFS) from healthy donors (Bahn *et al*., 2015). By RNA sequencing, Bahn *et al*. (2015) showed the presence of miRNAs, Piwi‐interacting RNAs, and circRNAs in CFS. It would be interesting to understand if the presence of these small RNAs in CFS is related to their interconnected mechanism of action. Interestingly, ontology analysis of the genes overlapping circRNAs indicates that salivary circRNAs are putatively involved in intercellular signaling and in inflammatory responses, opening the possibility of using saliva, an easily accessible body fluid, for detection of this new class of biomarkers of human diseases.

## 6. circRNA therapeutics in cancer: a glance at the molecular pathways of circPVT1

A growing number of studies showed that circRNAs play an important role in cancer. In fact, the same biochemical characteristics that make them suitable biomarkers may make circRNAs promising therapeutic targets. Interestingly, circRNAs can act in oncogenic molecular pathways involving different molecules, such as miRNAs, long noncoding RNAs, RNA‐binding proteins, and transcription factors. As an example, we discuss below our recent work on circPVT1 (Verduci *et al*., 2017). We showed that circPVT1, which comes from an exon of the long noncoding RNA PVT1, is a master regulator of HNSCC pathogenesis. Considering the upregulation of circPVT1 in cancer tissues, we assume that the splicing mechanism that generates circPVT1 from its host gene is preferred relative to other RNA splicing isoforms transcribed from the same gene, at least in the context of HNSCC oncogenesis. circPVT1 acts in HNSCC as a mediator of the oncogenic activities of mutant p53. Since TP53 is the most frequently mutated gene in human cancers (Kandoth *et al*., 2013), it is reasonable to theorize that circPVT1 could have a central role also in other type of cancer.

We showed that circPVT1 expression is transcriptionally enhanced by the mut‐p53/YAP/TEAD complex (Verduci *et al*., 2017). circPVT1 is also able to regulate its own expression and to recruit preferentially the mut‐p53/YAP/TEAD complex to its promoter region. To the best of our knowledge, it is the first example of how a circRNA is able to regulate its own expression level, opening a new perspective for the study of circRNAs’ mechanism of regulation. The oncogenic phenotype mediated by circPVT1 is explicated in HNSCC by its miRNA target, miR‐497‐5p. Note that our study did not exclude targeting of other miRNAs by circPVT1. Considering the tumor suppressor role of miR‐497‐5p in several cancers (Guo *et al*., 2013; Huang *et al*., 2015; Li *et al*., 2011; Wang *et al*., 2015), the finding that circPVT1 is able to sponge miR‐497‐5p opens a new avenue of research in other tumor types. The central roles played by circPVT1 in controlling the oncogenic phenotype, acting at both the transcriptional and post‐transcriptional levels, put this circRNA in a privileged position as a therapeutic target. Thus, not only the exceptional stability and high abundance of circRNAs make them promising therapeutic targets, but also their dominant functions in molecular pathways offer anticancer effect.

Up to now, different mechanisms of action have been attributed to different circRNAs. What remains poorly studied is the possibility that the same circRNAs can act in the same molecular pathway in different ways. We showed that circPVT1 acts both in the nucleus, by interacting with transcription factors and a nuclear cofactor, and in the cytoplasm, by sponging a specific miRNA. Presumably, additional circRNAs might employ more than one mode of action. Revealing the putative combined mechanisms of a circRNA would be very helpful for detailed characterization of oncogenic molecular pathways.

Although there are not yet any preclinical reports that use circRNAs as therapeutic targets for cancer treatment, this scenario is likely to change in the near future. Regarding circRNAs overexpressed in cancer, a possible therapeutic approach could be the use of siRNA against its sequence (Kristensen *et al*., 2018). Similarly to the approach adopted *in vitro* from us and others (Kristensen *et al*., 2018; Verduci *et al*., 2017), the designed siRNA should target the unique back‐splice junction of oncogenic circRNAs, avoiding any interference with the linear host gene. In the case of downregulated circRNAs in cancer, the restoration of the tumor suppressor circRNA level could be achieved by cloning the circRNA sequence and its regulatory flanking regions (Verduci *et al*., 2017). One of the main issues with circRNAs as a therapeutic approach is finding the best way to deliver *in vivo* the siRNAs or vectors expressing circRNA. Moreover, studies focusing on deeper characterization of circRNA biogenesis could be helpful to find a way to regulate their expression working on factors, such as splicing factors, responsible for circRNA biogenesis.

## 7. Concluding remarks

Gene expression is the result of multiple levels of regulation in both normal and pathological conditions. The noncoding part of the genome, represented by small and long noncoding RNAs, plays an important role in the regulation of gene expression in human diseases and especially in cancer. circRNAs and miRNAs are two of the main characters of this regulation (Aure *et al*., 2015; Stahlhut and Slack, 2013) (Fig. 3). circRNAs were considered in the past the outcome of transcriptional noise. Nowadays, they have become an important research subject, attracting interest from different areas, including the field of cancer research. As we described, circRNAs’ different mechanisms of action, their high stability, and their presence in accessible body fluids make circRNAs promising biomarkers and potential targets for therapeutic interventions, especially in personalized medicine. The study of the emerging circRNA–microRNA code will provide a novel understanding of molecular pathways involving pathogenesis‐related gene expression.

## Author contributions

LV and GB were major contributors in writing the manuscript. SS and YY edited the manuscript. YY edited the final version of all figures. All authors read, edited, and approved the final manuscript.

## Conflict of interest

The authors declare no conflict of interest.
